# The effectiveness of generic self‐management interventions for patients with chronic musculoskeletal pain on physical function, self‐efficacy, pain intensity and physical activity: A systematic review and meta‐analysis

**DOI:** 10.1002/ejp.1253

**Published:** 2018-06-27

**Authors:** S. Elbers, H. Wittink, J.J.M. Pool, R.J.E.M. Smeets

**Affiliations:** ^1^ Research Group Lifestyle and Health University of Applied Sciences Utrecht Utrecht The Netherlands; ^2^ Department of Rehabilitation Medicine CAPHRI School for Public Health and Primary Care Maastricht University Maastricht The Netherlands; ^3^ Libra Rehabilitation and Audiology Eindhoven/Weert The Netherlands

## Abstract

Generic self‐management programs aim to facilitate behavioural adjustment and therefore have considerable potential for patients with chronic musculoskeletal pain. Our main objective was to collect and synthesize all data on the effectiveness of generic self‐management interventions for patients with chronic musculoskeletal pain in terms of physical function, self‐efficacy, pain intensity and physical activity. Our secondary objective was to describe the content of these interventions, by means of classification according to the Behaviour Change Technique Taxonomy. We searched PubMed, CENTRAL, Embase and Psycinfo for eligible studies. Study selection, data extraction and risk of bias were assessed by two researchers independently. Meta‐analyses were only performed if the studies were sufficiently homogeneous and GRADE was used to determine the quality of evidence. We identified 20 randomized controlled trials that compared a self‐management intervention to any type of control group. For post‐intervention results, there was moderate quality evidence of a statistically significant but clinically unimportant effect for physical function and pain intensity, both favouring the self‐management group. At follow‐up, there was moderate quality evidence of a small clinically insignificant effect for self‐efficacy, favouring the self‐management group. All other comparisons did not indicate an effect. Classification of the behaviour change techniques showed large heterogeneity across studies. These results indicate that generic self‐management interventions have a marginal benefit for patients with chronic musculoskeletal pain in the short‐term for physical function and pain intensity and for self‐efficacy in the long‐term, and vary considerably with respect to intervention content.

**Significance:**

This study contributes to a growing body of evidence that generic self‐management interventions have limited effectiveness for patients with chronic musculoskeletal pain. Furthermore, this study has identified substantial differences in both content and delivery mode across self‐management interventions.

## 1 Introduction

Chronic musculoskeletal pain negatively influences daily life functioning, emotional well‐being and social participation (Turk et al., 2011). Low back pain and neck pain alone contribute to 1694 years lost to disability (YLD) per 100,000 persons annually, placing these conditions, respectively, first and fourth in the ranking of diseases on global years lived with disability (Vos et al., 2012).

The experience of pain interrupts individuals’ ongoing activities, forcing them to choose between pursuit of their intended action or activity, disengagement or avoidance behaviours. Motivational conflicts such as these constantly interfere with daily life activities and are assumed to have a negative effect on an individual's well‐being and identity (Vlaeyen et al., 2016). In order to maintain sufficient quality of life, successful self‐management – the ability to manage symptoms, treatment, physical, psychological and social consequences, and lifestyle changes related to one's chronic condition – is essential (Barlow et al., 2002; Lorig and Holman, 2003). To facilitate this process, generic self‐management interventions are designed to teach persons how to self‐regulate their chronic condition. Rather than providing unilateral solutions to disease‐specific problems, self‐management interventions provide a generic set of skills and competencies (e.g. problem‐solving, decision making, etc.) in order to facilitate living a meaningful life despite chronic pain.

The definition of self‐management does not specify how this behavioural adjustment should be achieved. This allows for a large variety of content and delivery modes in self‐management interventions. In order to provide more clarity in comparing interventions, Michie and colleagues have developed a taxonomy of behaviour change techniques (BCTs) that enables more precise reporting (Michie et al., 2013). Moreover, classification of components according to this taxonomy facilitates comparison of intervention content and is expected to provide insight into the various intended mechanisms of action.

As self‐management programmes aim to facilitate behavioural adjustment, they have considerable potential for positive long‐term effects on outcomes of importance to patients. However, as newly learned behaviours in the context of physical activity (Sullum et al., 2000) or pain rehabilitation (Turk and Rudy, 1991) are difficult to maintain, it is important to study the long‐term outcomes of these interventions.

### 1.1 The present study

Our primary aim was to collect and synthesize all available data on the immediate and long‐term (more than six months) effectiveness of generic self‐management interventions for patients with chronic musculoskeletal pain in terms of physical function, self‐efficacy, pain intensity and physical activity. We hypothesized that self‐management interventions would improve self‐efficacy, enabling patients with chronic pain to increase their physical activity, consequently reducing their perceived limitations in physical function, at least in the short‐term. Attributable to a shift in attention from disease‐related problems to engagement in daily life activities, patients might even perceive less pain after the intervention.

Our secondary aim was to describe the intervention content, by means of classification according to the Behaviour Change Technique Taxonomy (v1). This aim builds on recent efforts to acquire more insight into theory and techniques behind self‐management interventions (e.g. Keogh et al., 2015).

## 2 Method

### 2.1 Protocol and registration

The review protocol has been registered in the Prospero database (CRD42015024417).

### 2.2 Information sources

We searched MEDLINE via PubMed, CENTRAL, Embase and Psycinfo databases for eligible studies from inception up to May 2017. The search strategy was designed in collaboration with a medical informatics specialist and contained a combination of thesaurus terms and free text words. The PubMed search string (see Appendix S1) was constructed first and was used as a template for the other databases. The database search was extended in the following ways: first, reference lists of included articles were screened by one of the researchers (SE) and eligible studies underwent the same reviewing process (i.e. backward citation tracking). Second, when a study was included in the analysis, PubMed was used to search for eligible studies that cited this study (i.e. forward citation tracking). Third, to minimize publication bias, we also searched for unpublished studies and grey literature in DART‐Europe E‐thesis portal, the Open Access Thesis and Dissertations database (OATD), the WHO International Clinical Trials Registry Platform (WHO‐ICTRP), and the Networked Digital Library of Theses and Dissertations (NDLTD) with the combined terms ‘chronic pain’ and ‘self‐management’ as entry terms.

### 2.3 Eligibility criteria

We included randomized controlled trials that met the following eligibility criteria: The study sample had to consist of adult patients with chronic musculoskeletal pain, defined as pain that persists for longer than 3 months and that is perceived in the musculoskeletal system (i.e. bones, joints, tendons or muscles). Although self‐management principles have been incorporated in multicomponent treatment programmes (e.g. Meng et al., 2011; Du et al., 2017), and self‐management skill training can overlap with other types of interventions with different underlying theoretical approaches (e.g. action planning in the Health Action Process Approach, (Schwarzer, 2017), we were only interested in generic interventions that focused on improving behavioural adjustment by training self‐management skills. Therefore, the intervention had to address at least one of the following five self‐management skills: problem‐solving, decision making, resource utilization, forming a partnership with a health care provider and taking action (Lorig and Holman, 2003). In addition, the intervention had to include both an element of information transfer on self‐management principles (e.g. education session or lecture) and a training component where self‐management skills were actually rehearsed or performed. The intervention had to be focused on improving generic self‐management skills, rather than on training disease‐specific skills (e.g. joint protection techniques). The study had to include a control intervention that was not a self‐management intervention. Lastly, the study had to include at least one of the following outcome measures: physical function, self‐efficacy, pain intensity, or physical activity. For physical function, we included self‐report instruments that measured the degree of interference that chronic pain had on daily life activities and social participation. For self‐efficacy, we included self‐report instruments that measured the level of confidence in patients’ capabilities to perform daily life tasks or activities. Pain intensity measures were included if they solely measured the degree of pain experienced on a scale from low to high intensity. Composite scores of various moments of pain intensity were also included (e.g. Von Korff scales), as well as sum scores of pain intensity for each tender point. For physical activity, we included both self‐report instruments and activity trackers that provided an indication of how often certain types of physical activities were performed.

We excluded studies with samples that solely consisted of patients with osteoarthritis, because Kroon et al. (2014) had recently published a systematic review of self‐management interventions for this subgroup. When a composite sample included patients with osteoarthritis, at least 50% of the sample had to consist of patients with other forms of chronic musculoskeletal pain. In addition, interventions that were designed to improve self‐management in the context of pre‐operative training, post‐operative rehabilitation or palliative care were excluded, as we expected that this would lead to substantial heterogeneity regarding disease management and coping. To avoid heterogeneity, studies were also excluded if they only included patients on the basis of a specific comorbidity (e.g. psychiatric or obese patients), or if they combined the self‐management intervention with other chronic pain treatment modalities (e.g. graded activity, exposure in vivo, Acceptance and Commitment Therapy, interdisciplinary pain management programmes). We also excluded e‐health interventions that did not include any form of face‐to‐face contact during treatment, because a recent systematic review had been conducted on this topic (Eccleston et al., 2014). Only studies that were published in Dutch or English languages were included. We used the online application software ‘Rayyan’ to screen the abstracts (Ouzzani et al., 2016).

### 2.4 Study selection, data collection and risk of bias

Two researchers independently (HW and SE) performed the study selection, data collection and assessment of risk of bias in five stages. For each stage (abstract screening; full text inclusion; BCT data extraction; patient, intervention, comparison, outcome and study design data extraction; risk of bias assessment), we held pilot test sessions where we calibrated our procedures. At regular intervals within each stage, meetings were held to compare results and to reach consensus. If differences in scoring remained, a third researcher (JP) made the final decision. In the first stage, all abstracts were screened on eligibility criteria with respect to study design and patients. In the second stage, full text articles were read and checked on all eligibility criteria. Data collection started in the third stage and involved (1) copying all information regarding the intervention that was provided in the study or in the protocol; (2) extracting all individual intervention components from this information; and (3) classifying these components according to the BCT taxonomy v1 (Michie et al., 2013). In the fourth stage, we extracted all relevant data with respect to our analysis, including patient characteristics, means and standard deviations for all outcome measures of interest. For each study, we selected the measures that best fitted our definition for the primary outcomes. In accordance with the Cochrane Handbook, we considered studies as our primary source of interest (Higgins and Green, 2011). As a consequence, we also extracted data from study protocols and articles with follow‐up data, when they were available. In the fifth stage, we determined risk of bias using the Cochrane's Collaboration's tool for assessing risk of bias. The following types of bias were assessed: random sequence generation (selection bias); allocation concealment (selection bias); blinding of outcome assessment (detection bias); incomplete outcome data (attrition bias), selective reporting (reporting bias) and other sources of bias. Blinding of participants and personnel was not included in the bias assessment, as the characteristics of self‐management interventions do not allow for appropriate blinding. Due to the nature of the studies, we scored the default blinding of outcome assessment as high risk of bias, but upgraded to unclear or low if attempts to blind the outcome assessment for patients or assessors were described (e.g. blinding of patients to former assessment). All other types of bias were assessed according to the guidelines in the Cochrane Handbook (Higgins and Green, 2011). The risk of bias was used as input for the assessment of the quality of evidence for each outcome measure. Studies were considered high risk of bias when three or more items were scored unclear or high, or when two items were scored high.

### 2.5 Outcome reporting and data synthesis

Between‐group comparisons for post‐intervention (within one month of the end of the intervention) and follow‐up (at minimum six months post‐intervention) were calculated per study for each of the outcomes of interest, using RevMan 5.3 software (Cochrane, 2011). In case of more than one follow‐up measurement, we included the last time point in our analysis. If more than one self‐management group was included within a study, we used only the intervention group that best fitted our definition of self‐management interventions. In the situation of more than one control group within a study, we included only the most active control group in our comparisons. Results were presented for each outcome separately. If the GRADE analyses revealed both directness and consistency as a serious risk of bias, we concluded that the data were too heterogeneous to perform a meta‐analysis and presented the results narratively. Each outcome was expected to be measured with differing varying questionnaires. Therefore, standardized mean differences (SMD) with 95% confidence intervals were used. A priori, we decided to select random effects models because we assumed differences in the true outcomes across studies, based on between‐study variation in duration, intensity and patient characteristics. If the pooled SMD was significant, we re‐expressed this effect on one of the outcome measures to examine the clinical importance. This was performed by multiplying the SMD with the standard deviation of the control group of one of the included studies that adopted this measure. Subsequently, we compared this effect with available estimates of the minimal important change to assess the clinical importance. When it was not possible to obtain measures of central tendency or dispersion, the results were narratively presented and compared to the results of the meta‐analysis.

BCTs were graphically visualized in a table. Relative differences between studies and between domains of the taxonomy were calculated and presented narratively.

### 2.6 Assessment of the quality of evidence

For each comparison in the meta‐analysis, we used the GRADEpro Guideline Development Tool (Evidence Prime I, 2015) to determine the quality of evidence. As only randomized controlled trials were included, the initial quality of evidence started as ‘high’ and was downgraded as a result of limitations with respect to risk of bias, inconsistency, indirectness, imprecision or publication bias.

For each comparison, we downgraded the level of evidence when (1) more than 25% of the sample came from studies with high risk of bias; (2) the *I* ^2^ was more than 60% combined with a limited overlap of confidence intervals (inconsistency); (3) substantial differences were present in study population, intervention protocol, control group or outcome measures (indirectness); or (4) when the total sample size of all included studies was less than the optimal information size of *n *=* *400 (imprecision). We determined the optimal information size with a sample size calculation with α* *= 0.05, β* *= 0.8, SD* *= 0.2 as parameters (Schünneman et al., 2013). To assess publication bias, funnel plot symmetry and distribution of effect sizes were inspected. We based our quality of evidence criteria on the Grade Handbook (Schünneman et al., 2013) and the Cochrane Handbook (Higgins and Green, 2011).

## 3 Results

### 3.1 Study selection

**Figure 1:**
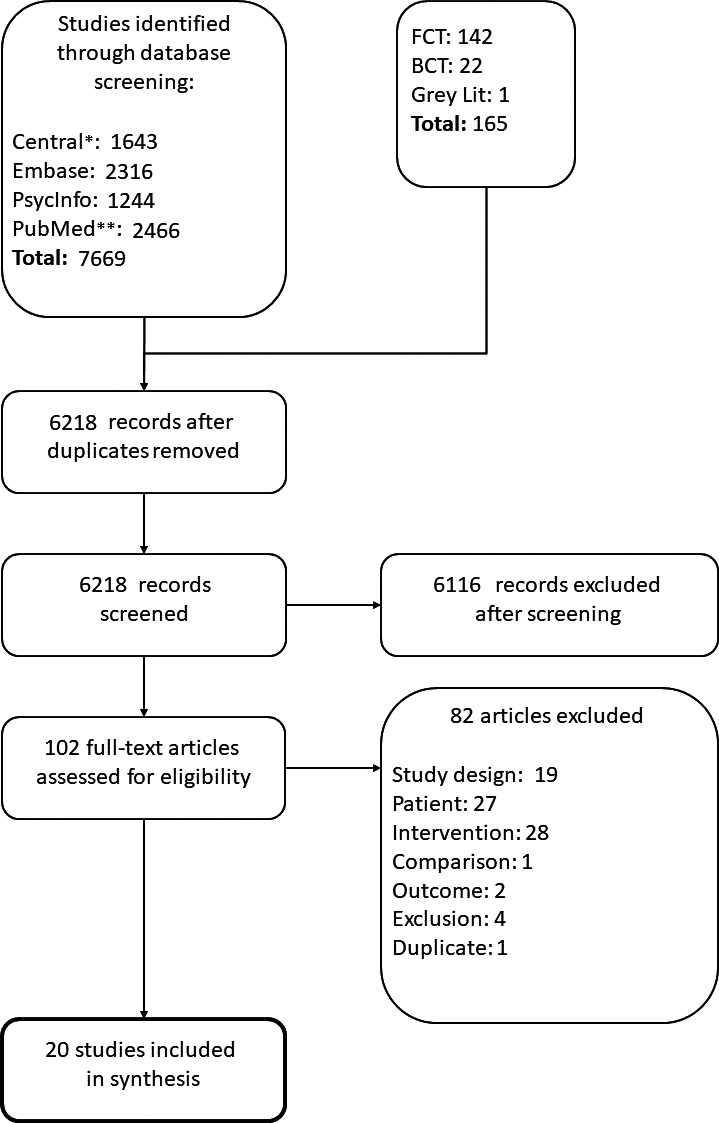
Flowchart of the literature search and study selection. *n* is the number of randomized controlled trials. FCT, forward citation tracking; BCT, backward citation tracking; Grey Lit, grey literature.

The search yielded 7843 hits. After removal of duplicates and the screening of abstracts, 102 full‐text articles were assessed for eligibility. Eighty‐two studies were excluded and 20 studies were selected for data extraction and analysis (see Fig. 1).

### 3.2 Patient and study characteristics

The total study population consisted of 3557 patients. Seventy‐five percent of the study population was female. All studies were performed in Western Europe, Australia or the United States. Average pain duration characteristics were only reported in eight studies and the means ranged from 2.3 to 20 years with a median of 8.4 years. Patient eligibility criteria varied across studies and were based on localization (e.g. back pain), specific diagnosis group (e.g. fibromyalgia syndrome), or duration of pain. Table 1 provides an overview of all participant characteristics within each study.

**Table 1:** Characteristics of participants within the included studies

Study	(*n*)	Age *M* (SD)	% female	Country	Pain duration, years *M* (SD)	Diagnosis group
Andersen et al. (2015)	141	45.23 (10.49)	55.71	Denmark	NR	Back pain or upper body pain
Arvidsson et al. (2013)	202	55.48 (12.05)	73	Sweden	NR	Rheumatic diseases
Asenlof et al. (2005)	122	42.56 (11.59)	77.3	Sweden	*Mdn *= 2.3^a^	Musculoskeletal pain
Burckhardt et al. (1994)	99	46.5 (8.3)	100	Sweden	7.5 (5.5)	Fibromyalgia syndrome
Dworkin et al. (2002)	124	37.7 (30.61)	84.68	United States	NR	Temporo‐mandibular disorders
Ersek et al. (2008)	256	81.85 (6.48)	84.75	United States	NR	Chronic pain (>3 months)
Gronning et al. (2012)	141	58 (11)	69	Norway	12 (13)	Polyarthritis
Haas et al. (2005)	120	77.2 (7.7)	84.4	United States	NR	Low back pain
Hutting et al. (2015)	123	46.24 (10.89)	75.9	Netherlands	NR	Chronic non‐specific CANS
King et al. (2002)	196	46.07 (9.05)	100	Canada	9.33 (9.22)	Fibromyalgia syndrome
Knittle et al. (2015)	78	62.75 (11.79)	66.69	Netherlands	NR	Rheumatoid arthritis
Lefort et al. (1998)	110	39.48 (25–60)^b^	75	Canada	6.07 (1–28)^b^	Non‐malignant chronic pain (>3 months)
Linton et al. (1997)	103	50.73 (9.71)	73.68	Sweden	NR	Musculoskeletal pain
Manning et al. (2014)	108	55.07 (15.55)	75.93	United Kingdom	20 (18.52)	Rheumatoid arthritis
Moore et al. (2000)	226	49.5 (10.6)	54	United States	NR	Back pain
Nicholas et al. (2013)	141	73.9 (6.5)	63	Australia	14.83 (17.3)	Chronic pain (>6 months)
Stuifbergen et al. (2010)	234	53.09 (9.86)	100	United States	NR	Fibromyalgia syndrome
Taal et al. (1993)	75	44.94 (24–64)^b^	73.68	Netherlands	4.3 (1–29)^b^	Rheumatoid arthritis
Taylor et al. (2016)	703	59.78 (13.67)	67	United Kingdom	NR	Chronic Musculoskeletal pain
Von Korff et al. (1998)	255	49.8 (11.3)	62.37	United States	NR	Back pain

NR, not reported.

a Median.

b Range.

The included studies show substantial variation regarding intervention content, delivery and measurement instruments (Table 2). For example, the median number of face‐to‐face sessions was 6 (range: 3–15), and the median duration was 15 h (range: 2.8–45 h). Furthermore, fifteen studies included a follow‐up measurement of at least six months post‐intervention, with a mean of 10.53 (SD* *= 2.59) months. The mean number of BCTs was 12.6 (range: 5–26). Forty‐three of the 93 available BCTs in the taxonomy were identified in the studies and we identified BCTs in all domains of the taxonomy, except for scheduled consequences and covert learning. The domains with the highest numbers of BCTs were goals and planning (accounting for 27.8% of the total BCTs), and social support (10.6%). Six BCTs were frequently used in the interventions: ‘Social support (unspecified) provided by group interventions’, ‘credible source provided by an experienced health care provider or patient’, and ‘goal setting (behaviour)’ were present in at least 90% of the interventions. ‘Problem‐solving’, ‘instruction on how to perform the behaviour’ and ‘information about health consequences (education)’ were present in 80–90% of the interventions. Appendix S2 provides a full overview of the BCT profiles per study.

Sixteen studies provided sufficient data to perform meta‐analyses. One of these studies used change scores to control for baseline differences (Manning et al., 2014), whereas the other studies used final value scores of their outcome measures. As both type of scores are not compatible within one calculation of a standardized mean difference, we analyzed the comparisons of Manning et al. (2014) separately. The four studies that could not be included in the meta‐analyses were presented narratively (Taal et al., 1993; Burckhardt et al., 1994; Dworkin et al., 2002; Hutting et al., 2015).

**Table 2:** Study characteristics of the included studies

Study	Study characteristics					Measurement characteristics				
	Setting	f2f sessions	Duration intervention^a^	Total BCT	Type of control	Physical activity	Physical function	Self‐efficacy	Pain intensity	Post and f/u measurements
Andersen et al. (2015, 2016)	NR	6 group sessions	15 h, within 6 weeks	10	1. Physical activity 2. Health guidance^d^				VAS^b^	1. f/u 3 month 2. f/u 11 month
Arvidsson et al. (2013)	NR	10 group sessions	15 h, within 1 year	8	Treatment as usual		SF‐36: PF			1. post 2. f/u 6 month
Asenlof et al. (2005)	Physical Therapy Clinic	8–10 sessions	Mean = 3.2 months	10	Exercise		PDI	SES	NRS^c^	1. post 2. f/u 3 month
Burckhardt et al. (1994)	NR	1. 6 group sessions 2. 12 group sessions^d^	1. 9 h, within 6 weeks 2. 15 h, within 6 weeks^d^	13	Waiting list		FIQ: Physical function	SES	Tender points	Post
Dworkin et al. (2002)	Pain clinic	3 group sessions	3 h 15 m within 2.5 months	15	Treatment as usual		Pain interference score		NRS^e^	1. post 2. f/u 6 month 3. f/u 12 month
Ersek et al. (2008)	Retirement facility	7 group sessions	10 h 30 m	14	Workbook		RDQ	ASES	NRS^e^	1. post 2. f/u 6 month 3. f/u 12 month
Gronning et al. (2012, 2013)	Hospital	3 sessions, 1 individual session	9 h 45 m	9	Treatment as usual		MHAQ		VAS	1. f/u 4 month 2. f/u 12 month
Haas et al. (2005)	Community building	6 group sessions	15 h within six weeks	13	Waiting list		MvK: disability		NRS^e^	f/u 6 month
Hutting et al. (2015)	NR	6 group sessions	15 h within 6 weeks	14	Treatment as usual		DASH	GES	NRS^f^	1. f/u 3 month 2. f/u 6 month 3. f/u 12 month
King et al. (2002)	University	1. Ed: 12 group sessions. 2. EdEx: 36 group sessions^d^	1. Ed: 21 h 2. EdEx: 31h^d^	11	1. Exercise 2. Workbook^d^			SES	Tenderpoints	1. post 2. f/u 3 month
Knittle et al. (2015)	Hospital	1 group session, 4 individual sessions	NR (Duration of individual sessions = 3 h 10 m) within 18 weeks	18	Education	SQuAsH	HAQ	Self‐efficacy for PA		1. post 2. f/u 6 month
Lefort et al. (1998)	Community setting	6 group sessions	12 h, within six weeks	5	Waiting list		1. SF‐36: PF 2.SOPA‐D^d^	SES		Post
Linton et al. (1997)	NR	15 group sessions	45 h, within 1 year	7	1. Emotional support 2. Treatment as usual^d^	MPI: general activity	SIP‐pain		MPI: pain severity	f/u 12 month
Manning et al. (2014)	Hospital	4 group sessions	4 h, within 12 weeks	16	Treatment as usual		DASH	ASES: function	VAS	1. post 2. f/u 6 mo
Moore et al. (2000)	NR	2 group sessions, 1 individual session.	2 h 48 m	6	Treatment as usual + self‐help book.		RDQ		NRS	1. f/u 3 month 2. f/u 6 month 3. f/u 12 month
Nicholas et al. (2013, 2017)	Pain clinic	8 group sessions	16 h, within 4 weeks	16	1. Exercise 2. waiting list^c^		RMDQ	PSEQ	NRS^f^	1. post 2. f/u 1 month 3. f/u 6 month 4. f/u 12 month
Stuifbergen et al. (2010)	NR	8 group sessions	16 h within 5 months	13	Active control: 8 classroom sessions + phone calls	HPLPII: physical activity	1. SF‐36: PCS 2. FIQ^d^	SRAHP		1. post 2. f/u 3 month 3. f/u 6 month
Taal et al. (1993)	NR	6 group sessions	15 h, within 6 weeks	15	Treatment as usual	AIMS: PA	MHAQ	ASES: function	AIMS: Pain	1. post 2. f/u 3 month 3. f/u 13 month
Taylor et al. (2016)	Community buildings; hospitals; hospices; university premises	3 day course	16 h15 m, within 3 weeks	26	Treatment as usual + book and relaxation CD		CPG	PSEQ	CPG: pain intensity	1. f/u 6 month 2. f/u 12 month
Von Korff et al. (1998)	NR	4 group sessions	8 h, within 4 weeks		Treatment as usual + book.		1. RDQ 2. NRS pain interferencesscore^d^		NRS^g^	1. f/u 3 month 2. f/u 6 month 3. f/u 12 month

AIMS, arthritis impact measuresment scales; ASES, arthritis self efficacy scale; CPG, chronic pain grade scale; DASH, disabilities of the arm shoulder and hand outcome measure; Ed, education; Ex, exercise; FIQ, fibromyalgia impact questionnaire; GES, general self‐efficacy scale; HAQ, health assessment questionnaire; HPLPII, health promoting lifestyle profileII; MHAQ, modified health assessment questionnaire; MPI, multidimensional pain inventory; MvK, modified von Korff scale; NR, not reported; NRS, numeric rating scale; PCS, physical component scale; PDI, pain disability index; RDQ, Roland Morris disability questionnaire; RMDQ, Roland Morris Disability Questionnaire; SES, self‐efficacy scale; SF‐36, short form 36; SIP, sickness impact profile; SOPA‐D, survey of pain attitudes, subscale disability; SquAsH, short questionnaire to assess health; SRAHP, self rated abilities for health practices scale; VAS, visual analogue scale.

a Hours of face to face contact.

b Average of the last seven days.

c NRS of average pain score.

d Not included in meta‐analysis.

e Average of NRS rating ‘pain right now’, ‘worst pain’ and ‘usual pain’.

f Pain in the previous week.

g Average pain intensity in the preceding 3 months.

### 3.3 Risk of bias

**Figure 2:**
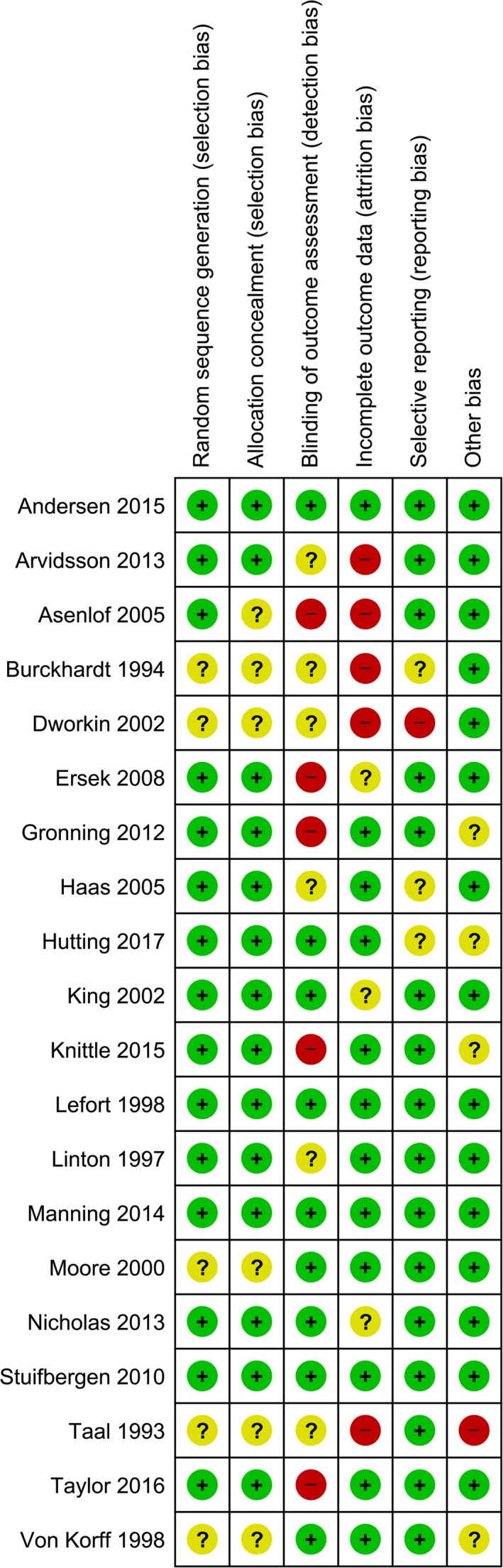
Risk of bias assessment of included studies.

The risk of bias was low for all studies, except for Asenlof et al. (2005), Taal et al. (1993), Dworkin et al. (2002), Burckhardt et al. (1994), and Von Korff et al. (1998). Fig. 2 shows all risk of bias assessments.

### 3.4 Immediate post‐intervention comparisons

#### 3.4.1 Physical function

**Figure 3:**
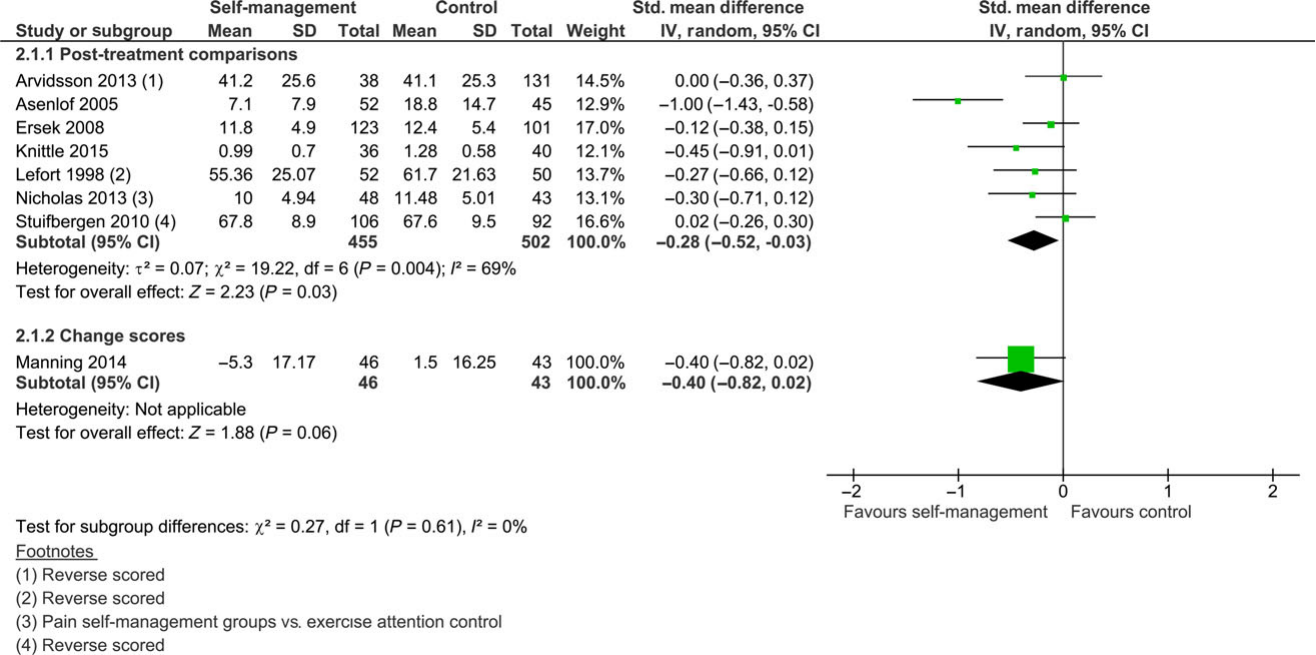
Post treatment comparison of self‐management intervention versus control on physical function.

For eight of the 11 studies that reported outcomes on physical function we were able to calculate standardized mean differences. Statistical pooling was considered appropriate despite a high *I* ^2^, as a sensitivity analysis revealed that the heterogeneity was mainly attributable to one study (Asenlof et al., 2005), and that the confidence intervals showed substantial overlap. The pooled effect was calculated with Hedges’ (adjusted) *g* and was significant; SMD −0.28 [−0.52, −0.03], *z *=* *2.23, *p *=* *0.03 (see Fig. 3). When this effect is re‐expressed on a Pain Disability Index (PDI), using the baseline standard deviation (SD* *= 14.7) of the control group of Asenlof et al. (2005), this effect corresponds to a between‐group difference of 4.12 points on a PDI favouring the self‐management group. This is lower than the minimal clinically important change of 8.5 points that was calculated by Soer et al. (2012). The between‐group comparison of post minus pre scores of Manning et al. (2014) was not significant; SMD −0.40 [−0.82, 0.02], *z *=* *1.88, *p = *0.06. Burckhardt et al. (1994) and Dworkin et al. (2002) reported no between‐group differences post‐intervention. Taal et al. (1993) found a statistically significant difference on the Modified Health Assessment Questionnaire (M‐HAQ), with the experimental group showing a mean decrease of 0.01 points and the control group showing a mean increase of 0.16 points compared to baseline scores (*p *<* *0.05).

#### 3.4.2 Self‐efficacy

**Figure 4:**
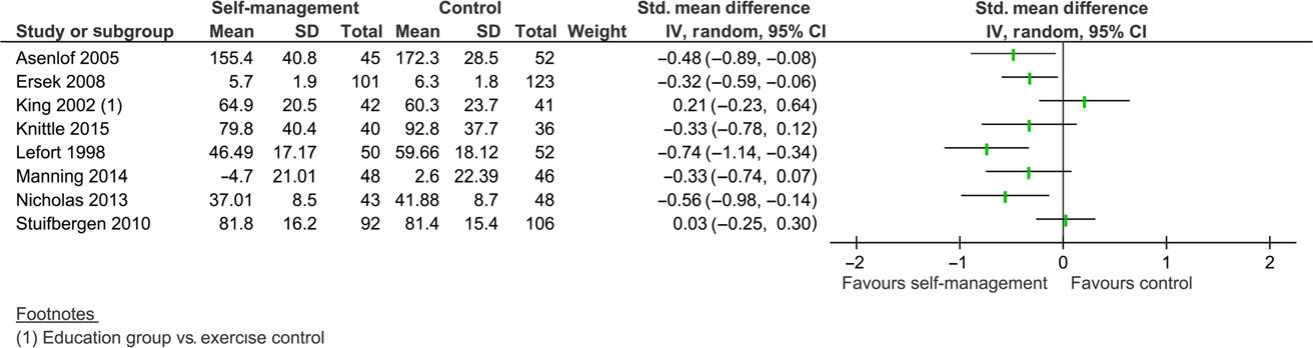
Post treatment comparison of self‐management intervention versus control on self‐efficacy.

Ten studies reported post‐intervention comparisons for self‐efficacy. However, due to the statistical heterogeneity (*I* ^2 ^= 65%, *χ* ^2^ (7965) = 17.39, *p *=* *0.02), an overall effect was not calculated. Four comparisons showed statistically significant differences, favouring the experimental group (Lefort et al., 1998; Asenlof et al., 2005; Ersek et al., 2008; Nicholas et al., 2013) with SMD ranging from −0.74 to −0.32. Furthermore, Taal et al. (1993) reported an effect favouring the self‐management intervention, with a change score of the experimental group (0.17) that significantly differed from the change score of the control group (−0.13), *p *<* *0.05. Burckhardt et al. (1994) also found a statistically significant difference between both groups, with the self‐management group reporting higher scores on the function subscale of the self‐efficacy scale (620.7), than the control group (467.5). Four comparisons did not show an effect, (King et al., 2002; Stuifbergen et al., 2010; Manning et al., 2014; Knittle et al., 2015). The unpooled comparisons, indicating a trend favouring self‐management, are shown in Fig. 4.

#### 3.4.3 Pain intensity

**Figure 5:**
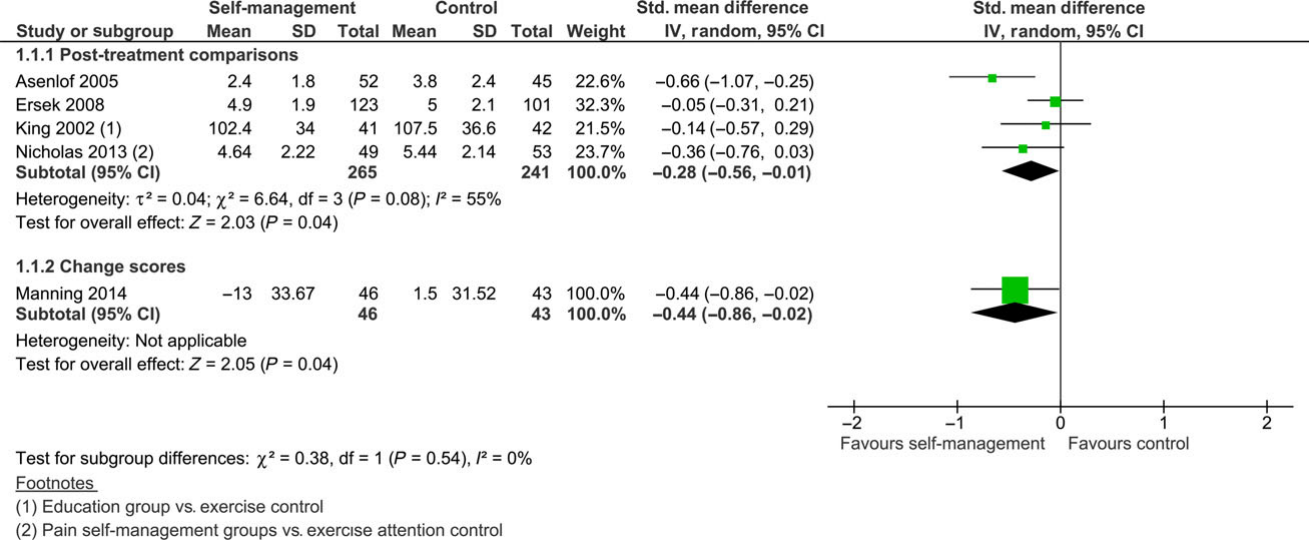
Post treatment comparison of self‐management intervention versus control on pain intensity.

Eight studies reported comparisons for pain intensity. Although the four studies that reported endpoint data showed substantial overlap of their confidence intervals, the *I* ^2^ was 55%. As a sensitivity analysis showed that the heterogeneity was contributable to only one study, a meta‐analysis was performed. The results indicate a statistically significant difference favouring the self‐management group, SMD −0.28 [−0.56, −0.01], *z *=* *2.03, *p *=* *0.04 (see Fig. 5). We re‐calculated this effect on an 11‐point NRS scale (0–10), using the baseline standard deviation of the control group in Nicholas et al. (2013). This effect corresponds to a 0.48 difference in pain intensity, measured on a 0–10 NRS, which is lower than the minimal clinically important difference (MCID) of 2.0 (Salaffi et al., 2004). Manning et al. (2014) reported a similar result: SMD −0.44 [−0.86, −0.02], *z *=* *2.05, *p *=* *0.04. Burckhardt et al. (1994), Dworkin et al. (2002) and Taal et al. (1993) reported no statistically significant post‐intervention differences for pain intensity between the self‐management and control groups.

#### 3.4.4 Physical activity

**Figure 6:**
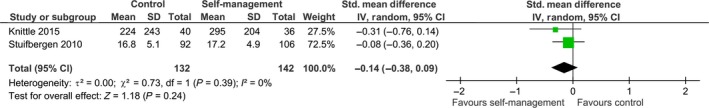
Post treatment comparison of self‐management intervention versus control on physical activity.

Only three studies compared differences in changes of physical activity immediately post‐treatment. Two studies provided sufficient information for a meta‐analysis (Stuifbergen et al., 2010; Knittle et al., 2015). We pooled these study outcomes as the *I* ^2^ was 0% and there was substantial overlap in the two confidence intervals. There was no significant difference between the intervention group and the control group, SMD 0.14 [0.38, −0.14], *z *=* *1.18, *p *=* *0.24 (see Fig. 6). This is in line with Taal et al. (1993), who also did not find a difference between self‐management and control groups on physical activity.

#### 3.4.5 Evaluation of the evidence

The GRADE evidence plot (Table 3) shows the post‐intervention comparisons combined with the quality of evidence. For each outcome measure, fewer than 25% of the participants were from high risk of bias studies. The inconsistency was high for self‐efficacy, due to high statistical heterogeneity compared with low overlap in confidence intervals. For the other outcome measures, the statistical heterogeneity was either limited or mainly contributable to one study. As a result of substantial variations in intervention content and outcome measures, all comparisons were downgraded for indirectness. Physical activity was the only comparison downgraded for imprecision, because the combined sample size was smaller than the optimal information size. Visual inspection of the funnel plots (see Appendix S3) did not indicate any publication bias. This resulted in the following evidence statements: For physical function and pain intensity, there is moderate quality evidence for a small but clinically insignificant effect favouring self‐management. For physical activity, there is low quality evidence for no effect of self‐management compared to a control group. Although we did not calculate standardized mean differences for self‐efficacy, based on the range of effects, we conclude that there is low quality evidence for a trend favouring the self‐management intervention. The studies that were not included in the meta‐analysis showed similar results and support these conclusions.

**Table 3:** GRADE evidence table for post‐intervention effects. Question: What is the short‐term effectiveness of self‐management interventions for patients with chronic pain compared to a control condition?

Quality assessment							Number of patients		Effect		Quality
Number of studies	Study design	Risk of bias	Inconsistency	Indirectness	Imprecision	Other considerations	Self‐management intervention	Control	Relative (95% CI)	Absolute (95% CI)	
Physical function (assessed with: questionnaires)											
8	Randomized trials	Not serious^a^	Not serious^b^^,^ c	Serious^d^^,^ e	Not serious^f^	None	501	545	‐	SMD 0.28 SD lower (0.52 lower to 0.03 lower)	⨁⨁⨁◯ MODERATE
Self efficacy (assessed with: questionnaires)											
8	Randomized trials	Not serious^a^	Serious^c^^,^ g	Serious^d^^,^ e	Not serious^f^	None	461	504	‐	Not pooled	⨁⨁◯◯ LOW
Pain intensity (assessed with: questionnaires)											
5	Randomized trials	Not serious^a^	Not serious^b^^,^ h	Serious^d^	Not serious^f^	None	311	248	‐	SMD 0.28 SD lower (0.56 lower to 0.01 lower)	⨁⨁⨁◯ MODERATE
Physical activity (assessed with: questionnaires; Scale from: 8 to 32)											
2	Randomized trials	Not serious^a^	Not serious^b^^,^ h	Serious^d^^,^ e	Serious^i^	None	142	132	‐	SMD 0.14 SD lower (0.38 lower to 0.09 higher)	⨁⨁◯◯ LOW

CI, confidence interval; SMD, standardized mean difference.

a Less than 25% of participants from high risk of bias studies.

b Substantial overlap in confidence intervals.

c I2 is more than 60%.

d Substantial differences in interventions.

e Substantial differences in outcome measures.

f Total sample size is more than optimal information size (alpha = 0.05; Beta = 0.2; ES = 0.2 SD).

g Limited overlap in confidence intervals.

h *I* ^2^ is less than 60%.

i Total sample size is less than optimal information size (alpha = 0.05; Beta = 0.2; ES = 0.2 SD).

### 3.5 Follow‐up results

#### 3.5.1 Limitations in physical function

**Figure 7:**
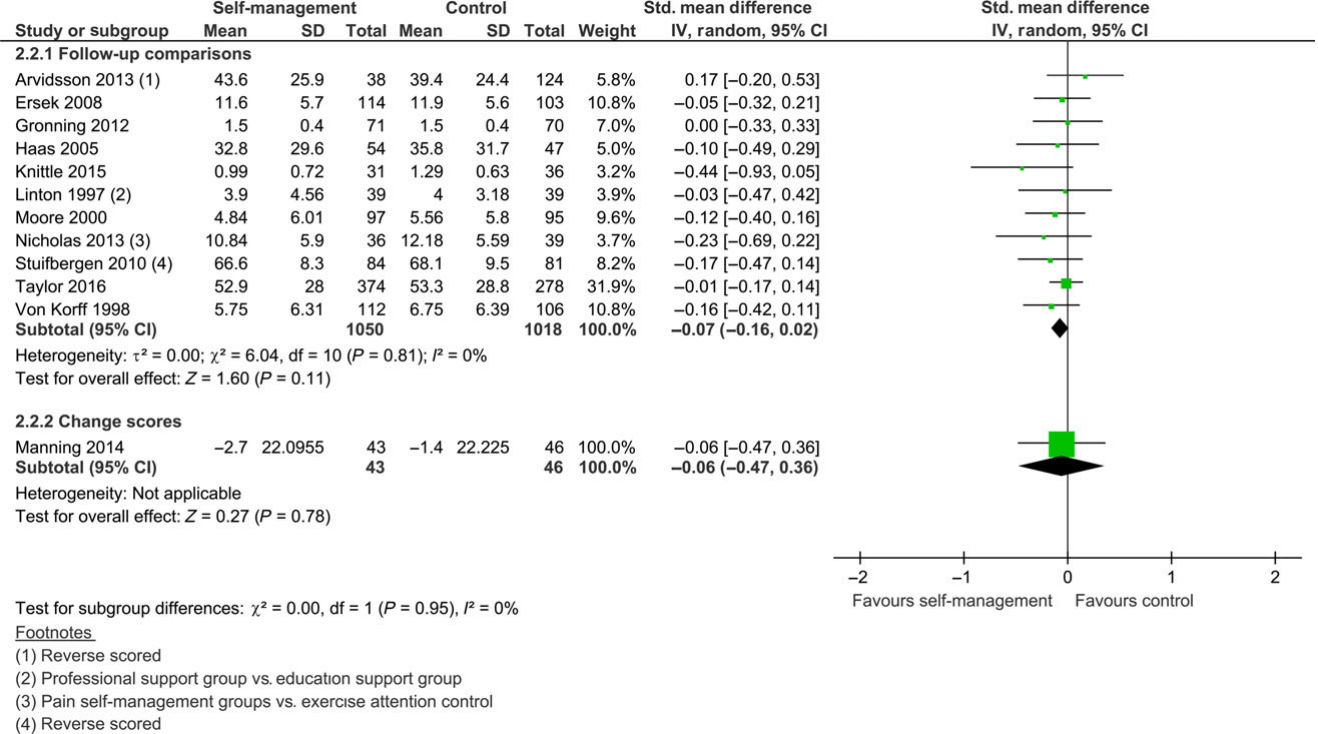
Follow‐up comparison of self‐management intervention versus control on physical function.

Twelve out of 15 studies with follow‐up data were eligible for pooling (see Fig. 7). The median follow‐up time of all 15 studies was 12 months. As the statistical heterogeneity was low (*I* ^2^ = 0%), we performed a meta‐analysis. The pooled effect of 11 studies with endpoint data was not statistically significant, SMD −0.07 [−0.16, 0.02], *z *=* *1.60, *p *=* *0.11, and this was also the case for Manning et al. (2014), SMD −0.06 [−0.47, 0.36], *z *=* *0.27, *p *=* *0.78. In addition, Hutting et al. (2015) and Taal et al. (1993) also reported no effects at follow‐up. The only study that reported a long‐term positive effect on physical function was Dworkin et al. (2002); at 12 months follow‐up, the self‐management group showed less limitation in physical function compared to control, *p *=* *0.01.

#### 3.5.2 Self‐efficacy

**Figure 8:**
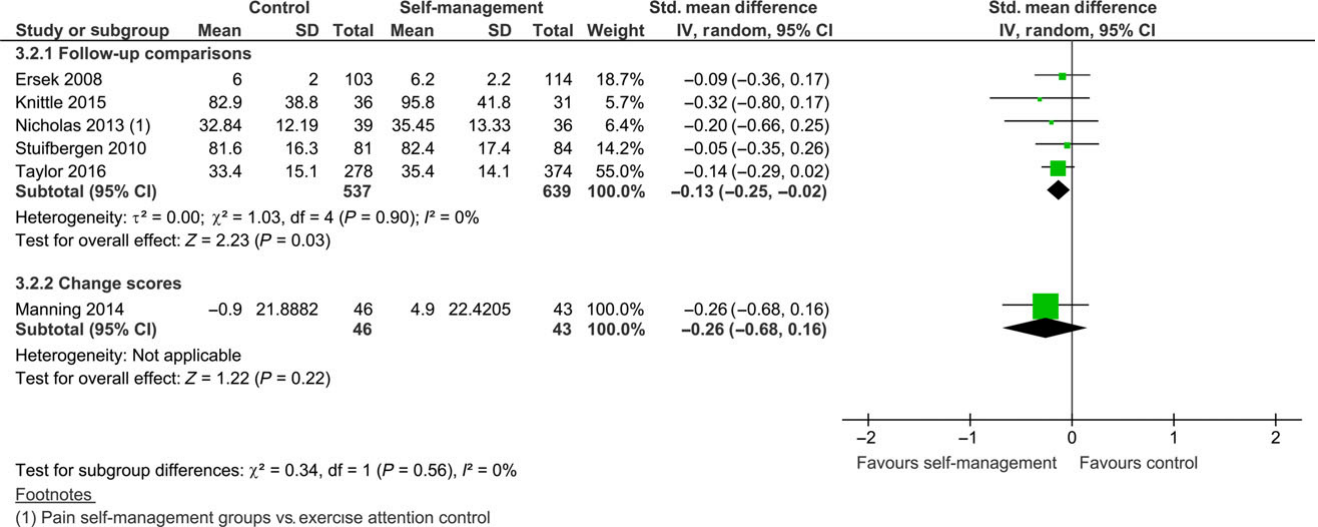
Follow‐up comparison of self‐management intervention versus control on self‐efficacy.

For self‐efficacy, the median follow‐up time was 12 months. Six of eight studies were included in the meta‐analysis (see Fig. 8). We found an *I* ^2^ of 0%, indicating homogeneous results across studies. For five studies with endpoint data, there was a significant effect for self‐efficacy at follow‐up favouring the self‐management group, SMD −0.13 [−0.25, −0.02], *z *=* *2.23, *p *=* *0.03. This corresponds to a difference of 1.72 points on the PSEQ (0–60), using the standard deviation of the baseline control group of Nicholas et al. (2013) as reference. As the minimal important change of the PSEQ is estimated at 5.5 points (Chiarotto et al., 2016), this mean difference is clinically insignificant. Manning et al. (2014) did not find a difference at follow‐up, SMD −0.26 [−0.68, 0.16], *z *=* *1.22, *p *=* *0.22. Taal et al. (1993) reported a significant difference for self‐efficacy favouring the self‐management group (*p *<* *0.05) with a positive change score of 0.17 for the self‐management group and a change score of −0.06 for the control group at 13 months follow‐up, whereas Hutting et al. (2015) did not find a difference at follow‐up.

#### 3.5.3 Pain intensity

**Figure 9:**
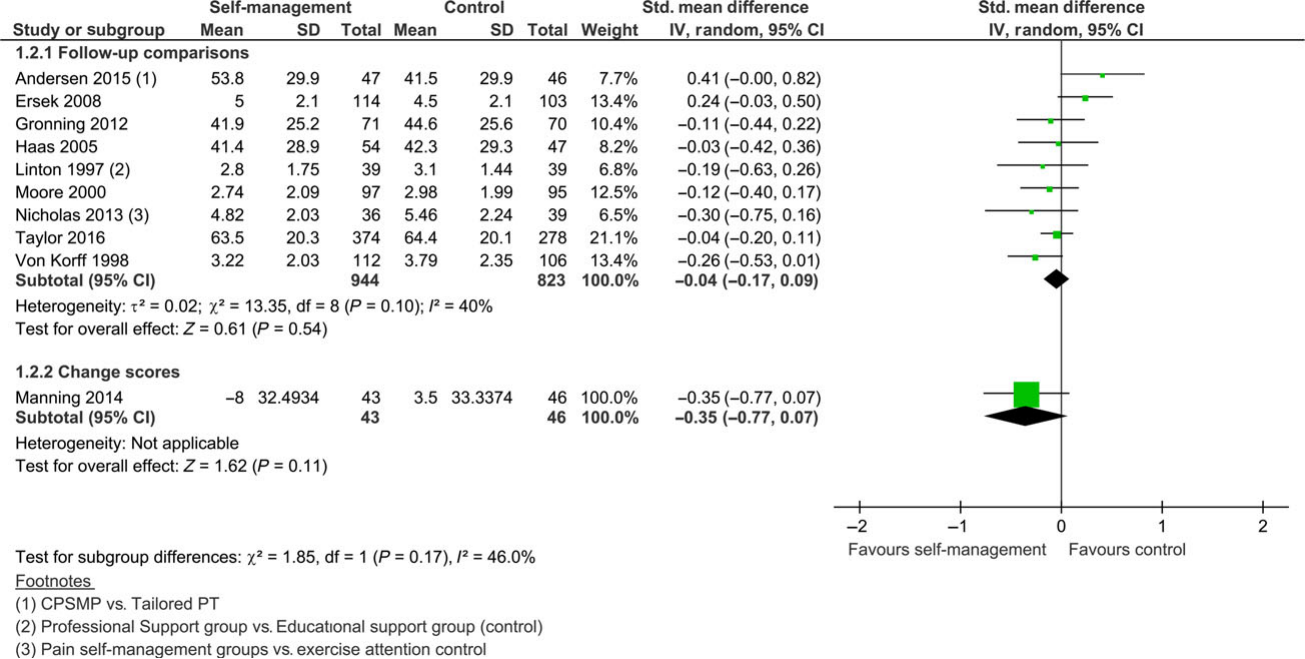
Follow‐up comparison of self‐management intervention versus control on pain intensity.

Ten of the 13 studies were included in the meta‐analysis (see Fig. 9). The median follow‐up time for all 13 studies was 12 months. We decided to pool the results as the *I* ^2^ was 40% and the overlap of confidence intervals was sufficient. The pooled standardized mean difference of the nine studies with endpoint data was not statistically significant, SMD −0.04 [−0.17, 0.09], *z *=* *0.61, *p *=* *0.54. This was similar for Manning et al. (2014): SMD −0.35 [−0.77, 0.07], *z *=* *1.62, *p *=* *0.11. Taal et al. (1993) and Hutting et al. (2015) also did not report a significant difference at follow‐up, but Dworkin et al. (2002) indicated that, at 12 months follow‐up, pain intensity was lower for the experimental group, compared to the control group (*p *=* *0.036).

#### 3.5.4 Physical activity

**Figure 10:**
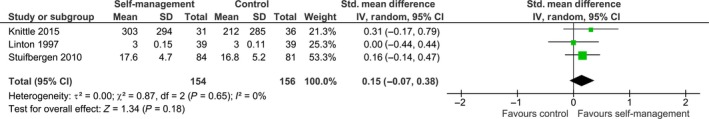
Follow‐up comparison of self‐management intervention versus control on physical activity.

Four studies provided information on follow‐up time for physical activity, with a median follow‐up time of 9 months. Three studies were eligible for pooling. We performed a meta‐analysis as the confidence intervals largely overlapped and the *I* ^2^ was 0% (see Fig. 10). There was no overall group‐effect on physical activity, SMD 0.15 [−0.07, 0.38], *z *=* *1.34, *p *=* *0.18. Taal et al. (1993) also reported no differences between both groups at follow‐up.

#### 3.5.5 Evaluation of the evidence

The GRADE evidence plot (Table 4) shows the standardized mean differences in combination with the quality of evidence ratings. The outcomes were evaluated similarly to the post‐intervention results, with the exception of a high consistency score for self‐efficacy. This resulted in the following evidence statements: At six to thirteen months follow‐up, there is moderate quality evidence that self‐management interventions have a statistically significant, but clinically unimportant effect on self‐efficacy. For pain and physical function, there is moderate quality evidence that self‐management intervention groups are not more effective than control groups. For physical activity, there is low quality evidence that self‐management interventions are not more effective than control groups. The studies that could not be included in the GRADE analysis showed similar trends and corroborated these conclusions.

**Table 4:** GRADE evidence table for follow‐up results. Question: What is the long‐term effectiveness of self‐management interventions for patients with chronic pain compared to a control condition?

Quality assessment							Number of patients		Effect		Quality
Number of studies	Study design	Risk of bias	Inconsistency	Indirectness	Imprecision	Other considerations	Self‐Management Intervention	Control	Relative (95% CI)	Absolute (95% CI)	
Physical function (follow‐up: range 6 months to 13 months; assessed with: questionnaires)											
12	Randomized trials	Not serious^a^	Not serious^b^^,^ c	Serious^d^^,^ e	Not serious^f^	None	1093	1064	‐	SMD 0.07 SD lower (0.16 lower to 0.02 higher)	⨁⨁⨁◯ MODERATE
Self‐efficacy (follow‐up: range 6 months to 13 months; assessed with: questionnaires)											
6	Randomized trials	Not serious^a^	Not serious^b^^,^ c	Serious^d^^,^ e	Not serious^f^	None	682	583	‐	SMD 0.13 SD lower (0.25 lower to 0.02 lower)	⨁⨁⨁◯ MODERATE
Pain (follow‐up: range 6 months to 13 months; assessed with: questionnaires)											
10	Randomized trials	Not serious^a^	Not serious^b^^,^ c	Serious^d^	Not serious^f^	None	987	869	‐	SMD 0.04 SD lower (0.17 lower to 0.09 higher)	⨁⨁⨁◯ MODERATE
Physical activity (follow up: range 8 months to 12 months; assessed with: questionnaires)											
3	Randomized trials	Not serious^a^	Not serious^b^^,^ c	Serious^d^^,^ e	Serious^g^	None	154	156	‐	SMD 0.15 SD higher (0.07 lower to 0.38 higher)	⨁⨁◯◯ LOW

CI, confidence interval; SMD, standardized mean difference.

a Less than 25% of participants from high risk of bias studies.

b Substantial overlap in confidence intervals.

c *I* ^2^ is less than 60%.

d Substantial differences in interventions.

e Substantial differences in outcome measures.

f Total sample size is more than optimal information size (alpha = 0.05, beta = 0.2, ES = 0.2 SD).

g Total sample size is less than optimal information size (alpha = 0.05; Beta = 0.2; ES = 0.2 SD).

## 4 Discussion

### 4.1 Summary of main results

The primary aim of this study was to investigate the effectiveness of self‐management interventions on physical function, self‐efficacy, pain intensity and physical activity for patients with chronic musculoskeletal pain. We identified 20 randomized controlled trials that compared a self‐management intervention to a control group. For post‐intervention results, we found moderate quality evidence for a statistically significant, but clinically unimportant effect on physical function and pain intensity, both favouring the self‐management group. We also found low quality evidence for a trend favouring self‐management interventions on self‐efficacy and for no effect on physical activity. There was moderate quality evidence for a small, clinically insignificant, effect on self‐efficacy at follow‐up. We found moderate quality evidence for no between‐group differences at follow‐up for the remaining outcome measures. The results from the meta‐analyses were corroborated by the studies that could not be included in the pooling. These findings indicate that self‐management interventions have an only marginal benefit for patients with chronic musculoskeletal pain both in the short and long‐term. Furthermore, we found a large variety in BCTs used, indicating substantial differences between interventions in how to teach self‐management skills.

### 4.2 Similarities to and differences with other systematic reviews

We identified four related systematic reviews that show similar trends in effectiveness. Jordan et al. (2010) identified one subgroup of self‐management interventions that specifically targeted patients with osteoarthritis. For both short‐ and long‐term comparisons with control groups, the effects on clinical outcomes were inconclusive: Three studies of seven showed improvement on pain intensity; and for functional disability and quality of life, one out of five studies reported better results for the self‐management group than the control group. In addition, Nolte and Osborne (2013) evaluated the outcomes of 18 self‐management interventions that adopted the Stanford criteria and concluded that these interventions were only marginally effective for pain, disability and depression. Only the median effect sizes for self‐efficacy, *d *=* *0.30 (Range: 0.05–0.72), and for knowledge, *d *=* *0.78 (range: −0.05 to 1.11), were medium to large at post‐intervention. Warsi et al. (2004) did not find a significant improvement on pain and disability associated with self‐management interventions for patients with arthritis. This also holds for Kroon et al. (2014), who performed a systematic review and meta‐analysis to assess the effectiveness of self‐management programmes in patients with osteoarthritis. They concluded that self‐management interventions caused small to no benefits, which is in line with the current findings. We also found two systematic reviews with contrasting findings. Du et al. (2011) studied self‐management interventions for patients with chronic musculoskeletal pain and concluded that these were effective on pain intensity and disability. However, the pooled results only showed a trend in favour of self‐management for patients with chronic low back pain and a statistically significant but small change in disability and pain intensity for patients with arthritis. For Du et al. (2017), the pooled comparisons (intervention vs. control) were statistically significant at all time points for patients with chronic low back pain, but the effect sizes were small (ranging from −0.20 to −0.29 for pain intensity and −0.19 to −0.28 for disability). Differences in inclusion criteria concerning the interventions could further explain the variations in outcomes.

### 4.3 Future directions

Although the results of this study may not be surprising in light of the previous findings from systematic reviews, there is a large body of evidence that shows how psychological adjustment in the situation of a chronic disease may lead to favourable outcomes, such as improved well‐being and adaptive lifestyle changes (Stanton et al., 2007; de Ridder et al., 2008; Kamper et al., 2015). Below we will discuss three ideas that may explain why generic self‐management interventions are not as effective as expected and that could direct future research and intervention design. First, lasting behaviour change is a daunting challenge, which involves not only motivational factors such as self‐efficacy and intention, but also automatic processes such as habit formation (Webb and Sheeran, 2006; Strack and Deutsch, 2004; Papies, 2016). For patients with enduring pain, these automatic factors may be of particular importance as they have coped with their pain often for several years, thereby allowing habitual routines to develop in response to pain perception. This could explain the marginal long‐term effects because habits are difficult to modify, especially when interventions do not take these automatic behavioural processes into account (Papies, 2016). In order to successfully counter these habitual behaviours in interventions, Papies (2016) proposes a different approach with more emphasis on analysing and modifying these specific routines. This personalized approach differs from generic self‐management interventions that provide one set of skills expected to benefit all patients. In order to capture the individual tailoring that is required in these interventions, we endorse the recommendation of Morley et al. (2013) to further explore the potential of single‐case methodology. For example, experience sampling technology – where multiple (near) real‐time self‐reports of thoughts, feelings or activities can be obtained – could provide a more detailed insight in longitudinal individual response patterns to treatment (Vlaeyen et al., 2001; Maes et al., 2015).

Second, Keogh et al. (2015) attribute the limited effectiveness and large variety in content and delivery of self‐management interventions to limited and inconsistent application of behaviour change theory throughout the intervention. Increased self‐efficacy is often mentioned as an explanatory (mediating) factor, but it remains unclear how more confidence in the capability to live a meaningful life with pain would explain all post‐intervention results, including pain intensity. In particular, as we only identified a post‐intervention trend for self‐efficacy favouring self‐management interventions, other mechanisms that have not yet been identified could be responsible for the small short‐term effects on pain intensity and physical function. We believe that future research on moderators and mediators of the relationship between self‐management interventions and outcome measures could provide insight in how to optimize the effectiveness of this type of intervention.

Third, despite the limited effectiveness of stand‐alone generic interventions, self‐management skills such as problem‐solving, action‐planning and decision making have the potential to reinforce existing pain management treatments. Indeed, self‐management is regarded as a common component in interdisciplinary pain management programmes and is expected to facilitate more active and resilient coping (McCracken and Turk, 2002; Turk et al., 2011). Future studies should investigate the interaction between self‐management skill training and disease‐specific treatment components. This would lead to more insights on the contribution of self‐management skill training to long‐term effects of pain management programmes.

### 4.4 Strengths and limitations

Although all included studies focused on enhancing generic self‐management skills in order to improve clinical outcomes, there was a large variation on how to achieve and measure this. As a consequence, the methodological heterogeneity of the included studies negatively influenced the robustness of the outcomes. Therefore, the overall quality of evidence was downgraded for each comparison on indirectness. This also caused us to select a random‐effects model, which made the pooled results difficult to interpret (Higgins and Green, 2011), even when the effect was re‐expressed on the measurement scale of interest. Although this method provides an indication of the clinical importance of the effect, it cannot be regarded as a conclusive result. This is mainly because MCIDs are concerned with the effect at individual patient level rather than on mean scores at group level. However, an advantage of statistical pooling over qualitative forms of synthesis is that sample weights are included in the calculation of the overall effect. Visual inspection of the forest plots showed that only few individual studies reported small but statistically‐significant effects, indicating that other forms of synthesis probably would have yielded similar interpretations. A second limitation is that our conclusions relate to average group effects and do not provide more detailed information on the proportion of patients that respond well to self‐management interventions. Although a responder analysis is recommended (Henschke et al., 2014), very few studies provided such details. The consequence is that we were unable to explore beyond an average effect at study level.

Furthermore, we aimed to expose the various mechanisms of self‐management interventions by identifying and classifying the behaviour change strategies as much as possible. This method revealed commonly used strategies (e.g. a focus on goals and planning) as well as variation in the selection of techniques to support adaptive behaviour change for patients with chronic pain. This approach opened the black box of self‐management interventions to a certain extent. Although it seemed a logical next step to investigate whether specific combinations of BCTs influence the outcomes (e.g. Michie et al., 2009), we refrained from doing these analyses. Due to the generally small standardized mean differences throughout the comparisons (range SMD between studies = −1 to 0.41), we hypothesized that further exploration would not yield meaningful information.

## 5 Conclusion

There is moderate quality evidence that generic self‐management interventions have a small clinically unimportant post‐intervention effect on physical function and pain intensity. For physical activity, there is low quality evidence for no post‐intervention effect and for self‐efficacy, though we identified a trend favouring self‐management interventions. At follow‐up, there is moderate quality evidence for no effect of self‐management interventions on physical function and pain, and low quality evidence for no effect on physical activity. In addition, we found a small but clinically unimportant long‐term effect for self‐management interventions on self‐efficacy. Overall, these findings indicate that self‐management interventions only have a marginal benefit on self‐efficacy, pain intensity, physical function, and physical activity for patients with chronic musculoskeletal pain.

## Supporting information


**Appendix S1.** Pubmed search strategy.

**Appendix S2.** Overview of behavior change techniques per study.

**Appendix S3.** Assessment of publication bias by means of funnel plots for each comparison.

## References

[ejp1253-bib-0001] Andersen, L.N. , Juul‐Kristensen, B. , Sorensen, T.L. , Herborg, L.G. , Roessler, K.K. , Sogaard, K. (2015). Efficacy of tailored physical activity or chronic pain self‐management programme on return to work for sick‐listed citizens: A 3‐month randomised controlled trial. Scand J Public Health 43, 694–703. 26113171 10.1177/1403494815591687

[ejp1253-bib-0002] Andersen, L.N. , Juul‐Kristensen, B. , Sorensen, T.L. , Herborg, L.G. , Roessler, K.K. , Sogaard, K. (2016). Longer term follow‐up on effects of tailored physical activity or chronic pain self‐management programme on return‐to‐work: A randomized controlled trial. J Rehab Med 48, 887–892. 10.2340/16501977-2159 27786344

[ejp1253-bib-0003] Arvidsson, S. , Bergman, S. , Arvidsson, B. , Fridlund, B. , Tingström, P. (2013). Effects of a self‐care promoting problem‐based learning programme in people with rheumatic diseases: A randomized controlled study. J Adv Nurs 69, 1500–1514. 22973890 10.1111/jan.12008

[ejp1253-bib-0004] Asenlof, P. , Denison, E. , Lindberg, P. (2005). Individually tailored treatment targeting activity, motor behavior, and cognition reduces pain‐related disability: A randomized controlled trial in patients with musculoskeletal pain. J Pain 6, 588–603. 16139778 10.1016/j.jpain.2005.03.008

[ejp1253-bib-0005] Barlow, J. , Wright, C. , Sheasby, J. , Turner, A. , Hainsworth, J. (2002). Self‐management approaches for people with chronic conditions: A review. Patient Educ Couns 48, 177–187. 12401421 10.1016/s0738-3991(02)00032-0

[ejp1253-bib-0006] Burckhardt, C.S. , Mannerkorpi, K. , Hedenberg, L. , Bjelle, A. (1994). A randomized, controlled clinical trial of education and physical training for women with fibromyalgia. J Rheumatol 21, 714–720. 8035399

[ejp1253-bib-0007] Chiarotto, A. , Vanti, C. , Cedraschi, C. , Ferrari, S. , de Lima e Sà Resende, F. , Ostelo, R.W. , Pillastrini, P. (2016). Responsiveness and minimal important change of the pain self‐efficacy questionnaire and short forms in patients with chronic low back pain. J Pain, 17, 707–718. 26975193 10.1016/j.jpain.2016.02.012

[ejp1253-bib-0008] Cochrane . (2014). Review Manager (RevMan) [Computer program] (Version 5.3). Copenhagen: The Nordic Cochrane Centre.

[ejp1253-bib-0009] Du, S. , Yuan, C. , Xiao, X. , Chu, J. , Qiu, Y. , Qian, H. (2011). Self‐management programs for chronic musculoskeletal pain conditions: A systematic review and meta‐analysis. Patient Educ Couns 85, e299–e310. 21458196 10.1016/j.pec.2011.02.021

[ejp1253-bib-0010] Du, S. , Hu, L. , Dong, J. , Xu, G. , Chen, X. et al. (2017). Self‐management program for chronic low back pain: A systematic review and meta‐analysis. Patient Educ Couns 100, 37–49. 27554077 10.1016/j.pec.2016.07.029

[ejp1253-bib-0011] Dworkin, S.F. , Huggins, K.H. , Wilson, L. , Mancl, L. , Turner, J. , Massoth, D. , Leresche, L. , Truelove, E. (2002). A randomized clinical trial using research diagnostic criteria for temporomandibular disorders‐axis II to target clinic cases for a tailored self‐care TMD treatment program. J Orofac Pain 16, 48–63. 11889659

[ejp1253-bib-0101] Eccleston, C. , Fisher, E. , Craig, L. , Duggan, G.B. , Rosser, B.A. , Keogh, E. (2014). Psychological therapies (internet‐delivered) for the management of chronic pain in adults. Cochrane Library 2, 1–58. 10.1002/14651858.CD010152.pub2 PMC6685592 24574082

[ejp1253-bib-0012] Ersek, M. , Turner, J.A. , Cain, K.C. , Kemp, C.A. (2008). Results of a randomized controlled trial to examine the efficacy of a chronic pain self‐management group for older adults. Pain 138, 29–40. 18086516 10.1016/j.pain.2007.11.003 PMC2536565

[ejp1253-bib-0013] Evidence Prime I . (2015). GRADEpro GDT: GRADEpro guideline development tool [Software]: McMaster University.

[ejp1253-bib-0014] Gronning, K. , Skomsvoll, J.F. , Rannestad, T. , Steinsbekk, A. (2012). The effect of an educational programme consisting of group and individual arthritis education for patients with polyarthritis ‐ a randomised controlled trial. Patient Educ Couns 88, 113–120. 22277625 10.1016/j.pec.2011.12.011

[ejp1253-bib-0015] Gronning, K. , Rannestad, T. , Skomsvoll, J.F. , Rygg, L.O. , Steinsbekk, A. (2013). Long‐term effects of a nurse‐led group and individual patient education programme for patients with chronic inflammatory polyarthritis ‐ a randomized controlled trial. J Clin Nurs 23, 1005–1017. 23875718 10.1111/jocn.12353

[ejp1253-bib-0016] Haas, M. , Groupp, E. , Muench, J. , Kraemer, D. , Brummel‐Smith, K. et al. (2005). Chronic disease self‐management program for low back pain in the elderly. J Manipulative Physiol Ther 28, 228–237. 15883575 10.1016/j.jmpt.2005.03.010

[ejp1253-bib-0017] Henschke, N. , Van Enst, A. , Froud, R. , Ostelo, R.W.G. (2014). Responder analyses in randomised controlled trials for chronic low back pain: An overview of currently used methods. Eur Spine J 23, 772–778. 24419902 10.1007/s00586-013-3155-0 PMC3960418

[ejp1253-bib-0018] Higgins, J.P.T. , Green, S. (2011). Cochrane Handbook for Systematic Reviews of Interventions Version 5.1.0 The Cochrane Collaboration.

[ejp1253-bib-0019] Hutting, N. , Staal, J.B. , Engels, J.A. , Heerkens, Y.F. , Detaille, S.I. , Nijhuis‐van der Sanden, M.W. (2015). Effect evaluation of a self‐management programme for employees with complaints of the arm, neck or shoulder: A randomised controlled trial. Occup Environ Med 72, 852–861. 26359220 10.1136/oemed-2015-103089

[ejp1253-bib-0020] Jordan, J.L. , Holden, M.A. , Mason, E.E. , Foster, N.E. (2010). Interventions to improve adherence to exercise for chronic musculoskeletal pain in adults (review). Cochrane Library 1, 1–64. 10.1002/14651858.CD005956.pub2 PMC6769154 20091582

[ejp1253-bib-0021] Kamper, S.J. , Apeldoorn, A.T. , Chiarotto, A. , Smeets, R.J. , Ostelo, R.W. , Guzman, J. , van Tulder, M.W. (2015). Multidisciplinary biopsychosocial rehabilitation for chronic low back pain: Cochrane systematic review and meta‐analysis. BMJ 350, h444. 25694111 10.1136/bmj.h444 PMC4353283

[ejp1253-bib-0022] Keogh, A. , Tully, M.A. , Matthews, J. , Hurley, D.A. (2015). A review of behaviour change theories and techniques used in group based self‐management programmes for chronic low back pain and arthritis. Man Ther 20, 727–735. 25865062 10.1016/j.math.2015.03.014

[ejp1253-bib-0023] King, S.J. , Wessel, J. , Bhambhani, Y. , Sholter, D. , Maksymowych, W. (2002). The effects of exercise and education, individually or combined, in women with fibromyalgia. J Rheumatol 29, 2620–2627. 12465163

[ejp1253-bib-0024] Knittle, K. , De Gucht, V. , Hurkmans, E. , Peeters, A. , Ronday, K. , Maes, S. , Vlieland, T.V. (2015). Targeting motivation and self‐regulation to increase physical activity among patients with rheumatoid arthritis: A randomised controlled trial. Clin Rheumatol 34, 231–238. 24213780 10.1007/s10067-013-2425-x

[ejp1253-bib-0025] Kroon, F.P. , der Van Burg, L.R. , Buchbinder, R. , Osborne, R.H. , Johnston, R.V. , Pitt, V. (2014). Self‐management education programmes for osteoarthritis *. Cochrane Library, 1*. 10.1002/14651858.CD008963.pub2 PMC11104559 24425500

[ejp1253-bib-0026] Lefort, S.M. , Gray‐Donald, K. , Rowat, K.M. , Jeans, M.E. (1998). Randomized controlled trial of a community‐based psychoeducation program for the self‐management of chronic pain. Pain 74, 297–306. 9520244 10.1016/s0304-3959(97)00190-5

[ejp1253-bib-0027] Linton, S.J. , Hellsing, A.L. , Larsson, I. (1997). Bridging the gap: Support groups do not enhance long‐term outcome in chronic back pain. Clin J Pain 13, 221–228. 9303254 10.1097/00002508-199709000-00007

[ejp1253-bib-0028] Lorig, K.R. , Holman, H.R. (2003). Self‐management education: History, definition, outcomes and mechanisms. Ann Behav Med 26, 1–7. 12867348 10.1207/S15324796ABM2601_01

[ejp1253-bib-0029] Maes, I.H.L. , Delespaul, P.A.E.G. , Peters, M.L. , White, M.P. , van Horn, Y. , Schruers, K. , Anteunis, L. , Joore, M. (2015). Measuring health‐related quality of life by experiences: The experience sampling method. Value Health 18, 44–51. 25595233 10.1016/j.jval.2014.10.003

[ejp1253-bib-0030] Manning, V.L. , Hurley, M.V. , Scott, D.L. , Coker, B. , Choy, E. , Bearne, L.M. (2014). Education, self‐management, and upper extremity exercise training in people with rheumatoid arthritis: A randomized controlled trial. Arthr Care Res 66, 217–227. 10.1002/acr.22102 23925924

[ejp1253-bib-0031] McCracken, L.M. , Turk, D.C. (2002). Behavioral and cognitive‐behavioral treatment for chronic pain. Spine 27, 2564–2573. 12435995 10.1097/00007632-200211150-00033

[ejp1253-bib-0102] Meng, K. , Seekatz, B. , Roband, H. , Worringen, U. , Vogel, H. , Faller, H. (2011). Intermediate and long‐term effects of a standardized back school for inpatient orthopedic rehabilitation on illness knowledge and self‐management behaviors: a randomized controlled trial. Clin J Pain 27, 248–257. 21178600 10.1097/AJP.0b013e3181ffbfaf

[ejp1253-bib-0032] Michie, S. , Abraham, C. , Whittington, C. , McAteer, J. , Gupta, S. (2009). Effective techniques in healthy eating and physical activity interventions: A meta‐regression. Health Psychol 28, 690–701. 19916637 10.1037/a0016136

[ejp1253-bib-0033] Michie, S. , Richardson, M. , Johnston, M. , Abraham, C. , Francis, J. et al. (2013). The behavior change technique taxonomy (v1) of 93 hierarchically clustered techniques: Building an international consensus for the reporting of behavior change interventions. Ann Behav Med 46, 81–95. 23512568 10.1007/s12160-013-9486-6

[ejp1253-bib-0034] Moore, J.E. , Von Korff, M. , Cherkin, D. , Saunders, K. , Lorig, K. (2000). A randomized trial of a cognitive‐behavioral program for enhancing back pain self care in a primary care setting. Pain 88, 145–153. 11050369 10.1016/S0304-3959(00)00314-6

[ejp1253-bib-0035] Morley, S. , Williams, A. , Ecclestone, C. (2013). Examining the evidence about psychological treatments for chronic pain: Time for a paradigm shift? Pain 154, 1929–1931. 23742793 10.1016/j.pain.2013.05.049

[ejp1253-bib-0036] Nicholas, M.K. , Asghari, A. , Blyth, F.M. , Wood, B.M. , Murray, R. et al. (2013). Self‐management intervention for chronic pain in older adults: A randomised controlled trial. Pain 154, 824–835. 23522927 10.1016/j.pain.2013.02.009

[ejp1253-bib-0037] Nicholas, M.K. , Asghari, A. , Blyth, F.M. , Wood, B.M. , Murray, R. et al. (2017). Long‐term outcomes from training in self‐management of chronic pain in an elderly population: A randomized controlled trial. Pain 158, 86–95. 27682207 10.1097/j.pain.0000000000000729

[ejp1253-bib-0038] Nolte, S. , Osborne, R.H. (2013). A systematic review of outcomes of chronic disease self‐management interventions. Qual Life Res 22, 1805–1816. 23111571 10.1007/s11136-012-0302-8

[ejp1253-bib-0039] Ouzzani, M. , Hammady, H. , Fedorowicz, Z. , Elmagarmed, A. (2016). Rayyan ‐ a web and mobile app for systematic reviews. Syst Rev 5, 210. 27919275 10.1186/s13643-016-0384-4 PMC5139140

[ejp1253-bib-0103] Papies, E.K. (2016). Health goal priming as a situated intervention tool: how to benefit from nonconscious motivational routes to health behaviour. Health Psychol Rev 10, 408–424. 27144729 10.1080/17437199.2016.1183506 PMC5214881

[ejp1253-bib-0040] de Ridder, D.T.D. , Geenen, R. , Kuijer, R. , van Middendorp, H. (2008). Psychological adjustment to chronic disease. Lancet 372, 246–255. 18640461 10.1016/S0140-6736(08)61078-8

[ejp1253-bib-0041] Salaffi, F. , Stancati, A. , Silvestri, C.A. , Ciapetti, A. , Grassi, W. (2004). Minimal clinically important changes in chronic musculoskeletal pain intensity measured on a numerical rating scale. Eur J Pain 8, 283–291. 15207508 10.1016/j.ejpain.2003.09.004

[ejp1253-bib-0042] Schünneman, H. , Brozek, J. , Guyatt, G. , Oxman, A. (2013). GRADE Handbook for grading quality of evidence and strength of recommendations.

[ejp1253-bib-0104] Schwarzer, R. (2008). Modeling health behavior change: How to predict and modify the adoption and maintenance of health behaviors. Appl Psychol 57, 1–29.

[ejp1253-bib-0043] Soer, R. , Reneman, M.F. , Vroomen, P.C.A.J. , Stegeman, P. , Coppes, M.H. (2012). Responsiveness and minimal clinically important change of the pain disability index in patients with chronic back pain. Spine 37, 711–715. 21796022 10.1097/BRS.0b013e31822c8a7a

[ejp1253-bib-0044] Stanton, A.L. , Revenson, T.A. , Tennen, H. (2007). Health psychology: Psychological adjustment to chronic disease. Ann Rev Psychol 58, 565–592. 16930096 10.1146/annurev.psych.58.110405.085615

[ejp1253-bib-0105] Strack, F. , Deutsch, R. (2004). Reflective and impulsive determinants of social behavior. Pers Soc Psychol Rev 8, 220–247. 15454347 10.1207/s15327957pspr0803_1

[ejp1253-bib-0045] Stuifbergen, A.K. , Blozis, S.A. , Becker, H. , Phillips, L. , Timmerman, G. , Kullberg, V. , Morrison, J. (2010). A randomized controlled trial of a wellness intervention for women with fibromyalgia syndrome. Clin Rehabil 24, 305–318. 20360151 10.1177/0269215509343247 PMC7236616

[ejp1253-bib-0046] Sullum, J. , Clark, M.M. , King, T.K. (2000). Predictors of exercise relapse in a college population. J Am Coll Health 48, 175–180. 10650735 10.1080/07448480009595693

[ejp1253-bib-0047] Taal, E. , Riemsma, R.P. , Brus, H.L. , Seydel, E.R. , Rasker, J.J. , Wiegman, O. (1993). Group education for patients with rheumatoid arthritis. Patient Educ Couns 20, 177–187. 8337194 10.1016/0738-3991(93)90131-f

[ejp1253-bib-0048] Taylor, S.J.C. , Carnes, D. , Homer, K. , Pincus, T. , Kahan, B.C. et al. (2016). Improving the self‐management of chronic pain: COping with persistent Pain, Effectiveness Research in Self‐management (COPERS). In *Improving the self‐management of chronic pain: COping with persistent Pain, Effectiveness Research in Self‐management (COPERS)*. Southampton (UK).

[ejp1253-bib-0049] Turk, D.C. , Rudy, T.E. (1991). Neglected topics in the treatment of chronic pain patients ‐ relapse, noncompliance, and adherence enhancement. Pain 44, 5–28. 2038489 10.1016/0304-3959(91)90142-K

[ejp1253-bib-0050] Turk, D.C. , Wilson, H.D. , Cahana, A. (2011). Treatment of chronic non‐cancer pain. Lancet 377, 2226–2235. 21704872 10.1016/S0140-6736(11)60402-9

[ejp1253-bib-0051] Vlaeyen, J.W. , de Jong, J. , Geilen, M. , Heuts, P.H.T.G. , van Breukelen, G. (2001). Graded exposure in vivo in the treatment of pain‐related fear: A replicated single‐case experimental design in four patients with chronic low back pain. Behav Res Ther 39, 151–166. 11153970 10.1016/s0005-7967(99)00174-6

[ejp1253-bib-0052] Vlaeyen, J.W. , Morley, S. , Crombez, G. (2016). The experimental analysis of the interruptive, interfering, and identity‐distorting effects of chronic pain. Behav Res Ther 86, 23–34. 27614948 10.1016/j.brat.2016.08.016

[ejp1253-bib-0053] Von Korff, M. , Moore, J.E. , Lorig, K.R. , Cherkin, D. , Saunders, K. et al. (1998). A randomized trial of a lay person‐led self‐management group intervention for back pain patients in primary care. Spine 23, 2608–2615. 9854760 10.1097/00007632-199812010-00016

[ejp1253-bib-0054] Vos, T. , Flaxman, A.D. , Naghavi, M. , Lozano, R. , Michaud, C. et al. (2012). Years lived with disability (YLDs) for 1160 sequelae of 289 diseases and injuries 1990‐2010: A systematic analysis for the Global Burden of Disease Study 2010. Lancet 380, 2163–2196. 23245607 10.1016/S0140-6736(12)61729-2 PMC6350784

[ejp1253-bib-0055] Warsi, A. , Wang, P.S. , LaValley, M.P. , Avorn, J. , Solomon, D. (2004). Self‐management education programs in chronic disease. Arch Intern Med 164, 1641–1649. 15302634 10.1001/archinte.164.15.1641

[ejp1253-bib-0056] Webb, T.L. , Sheeran, P. (2006). Does changing behavioral intentions engender behavior change? A meta‐analysis of the experimental evidence. Psychol Bull 132, 249–268. 16536643 10.1037/0033-2909.132.2.249

