# Supplementary material for: The effectiveness of generic self‐management interventions for patients with chronic musculoskeletal pain on physical function, self‐efficacy, pain intensity and physical activity: A systematic review and meta‐analysis
Source: Eur J Pain. 2018 Jun 27;22(9):1577–96. doi: 10.1002/ejp.1253 (PMC6175326; doi:10.1002/ejp.1253)
Supplement: Supplementary file 1 — Appendix S1. Pubmed search strategy. [file EJP-22-1577-s001.docx]

**Appendix 1.** Pubmed search strategy.

| Review title | The Effectiveness of Self-Management Interventions on Self-Efficacy, Physical Activity and Disability for Patients with Chronic Musculoskeletal Pain: a Systematic Review |
| --- | --- |
| Search string details | Search strategy for MEDLINE database. The categories ‘patient’ and ‘intervention’ are combined with “AND”. Filter trial type: Clinical trials  This string will serve as a template for the search strings in the PsycINFO, EMBASE and CENTRAL database. |
| **Patient** | |
| (("Chronic pain"[Mesh] OR "Fibromyalgia"[Mesh] OR "Whiplash Injuries"[Mesh] OR "Cumulative Trauma Disorders"[Mesh] OR "Arthritis"[Mesh] OR "chronic pain"[tiab] OR "chronic pains"[tiab] OR "neuralgia"[tiab] OR "fibromyalgia"[tiab] OR "fibromyalgias"[tiab] OR "fibrositis"[tiab] OR "muscular rheumatism"[tiab] OR "whiplash"[tiab] OR "repetitive strain injury"[tiab] OR "repetitive strain injuries"[tiab] OR "repetition strain injury"[tiab] OR "repetition strain injuries"[tiab] OR "overuse injury"[tiab] OR "overuse injuries"[tiab] OR "overuse syndrome"[tiab] OR "cumulative trauma disorder"[tiab] OR “repetitive motion disorder”[tiab] OR “repetitive motion disorders”[tiab] OR "cumulative trauma disorders"[tiab] OR "arthritis"[tiab] OR "arthrides"[tiab] OR "osteoarthritis"[tiab] OR "osteoarthritides"[tiab] OR "osteoarthosis"[tiab] OR "osteoarthroses"[tiab] OR "arthralgia"[tiab] OR "arthralgias"[tiab])) | |
| **Intervention** | |
| (("Patient Education as Topic"[Mesh] OR “education of patients”[tiab] OR “patient education”[tiab] OR training[tiab] OR "Rehabilitation"[Mesh] OR "rehabilitation" [Subheading] OR rehabilitation[tiab]) AND ("Self Efficacy"[Mesh] OR “Health Behavior”[mesh] OR "Illness Behavior"[Mesh] OR "Goals"[Mesh] OR "Life Style"[Mesh:NoExp] OR "Attitude to Health"[Mesh] OR "Adaptation, Psychological"[Mesh] OR "Exercise"[Mesh] OR "Social Support"[Mesh] OR "Patient Participation"[Mesh] OR self efficacy[tiab] OR health behavior*[tiab] OR health behaviour*[tiab] OR illness behavior*[tiab] OR illness behaviour*[tiab] OR sickness behavior*[tiab] OR sickness behaviour*[tiab] OR goal[tiab] OR goals[tiab] OR life style[tiab] OR life styles[tiab] OR attitude to health[tiab] OR attitudes to health[tiab] OR health attitude[tiab] OR health attitudes[tiab] OR proactive coping[tiab] OR psychological adjustment[tiab] OR psychological adjustments[tiab] OR psychologic adaptation[tiab] OR psychologic adaptations[tiab] OR adaptive behavior*[tiab] OR adaptive behaviour*[tiab] OR self regulation[tiab] OR social support[tiab] OR social network OR patient participation[tiab] OR empower*[tiab]) OR “self management”[tiab] OR “Self Care”[MESH] OR “self care”[tiab]) | |
