# Supplementary material for: The effectiveness of generic self‐management interventions for patients with chronic musculoskeletal pain on physical function, self‐efficacy, pain intensity and physical activity: A systematic review and meta‐analysis
Source: Eur J Pain. 2018 Jun 27;22(9):1577–96. doi: 10.1002/ejp.1253 (PMC6175326; doi:10.1002/ejp.1253)
Supplement: Supplementary file 3 — Appendix S3. Assessment of publication bias by means of funnel plots for each comparison. [file EJP-22-1577-s003.docx]

**Appendix 3.** Assessment of publication bias by means of funnel plots for each comparison.


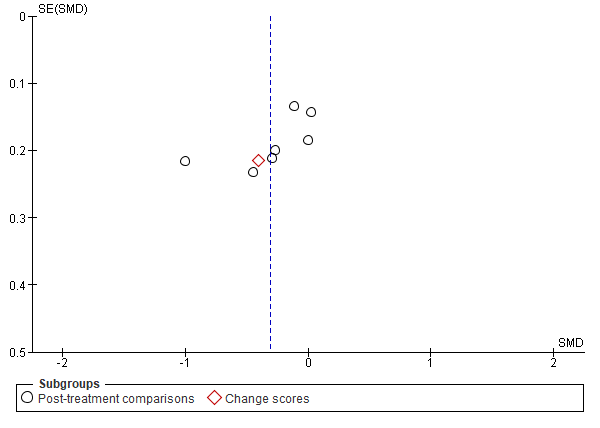


**Figure 1.** Funnel plot of comparison: 1 Physical function, post treatment.


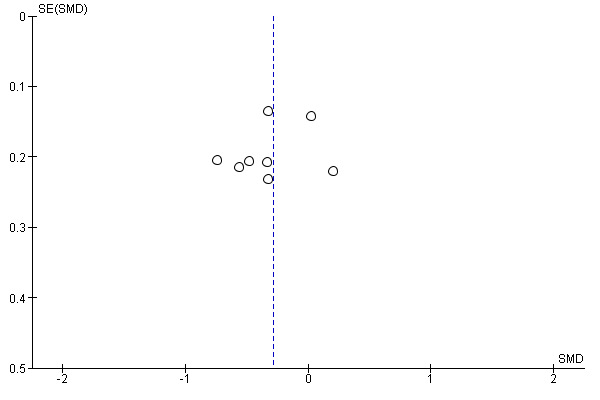


**Figure 2.** Funnel plot of comparison: 2 Self-efficacy, post treatment.


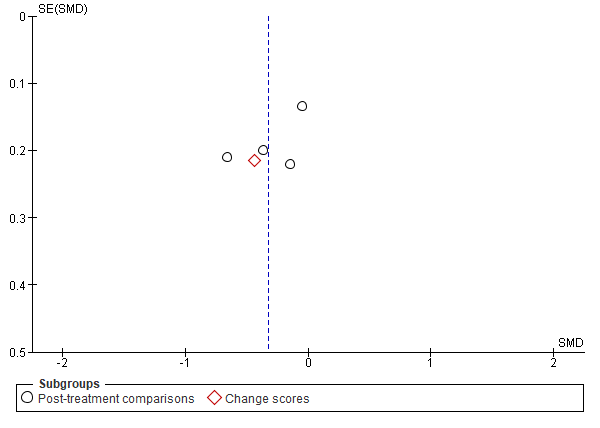


**Figure 3.** Funnel plot of comparison: 3 Pain intensity, post treatment.


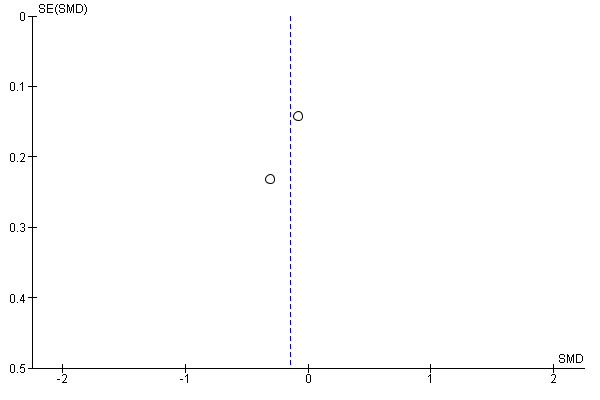


**Figure 4.** Funnel plot of comparison: 4 Physical activity, post treatment.


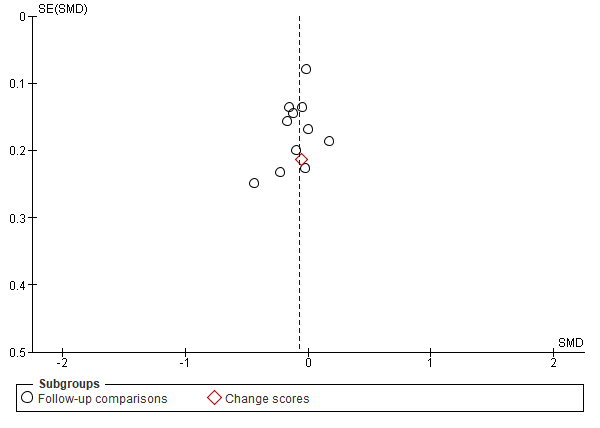


**Figure 5.** Funnel plot of comparison: 5 Physical function, follow-up.


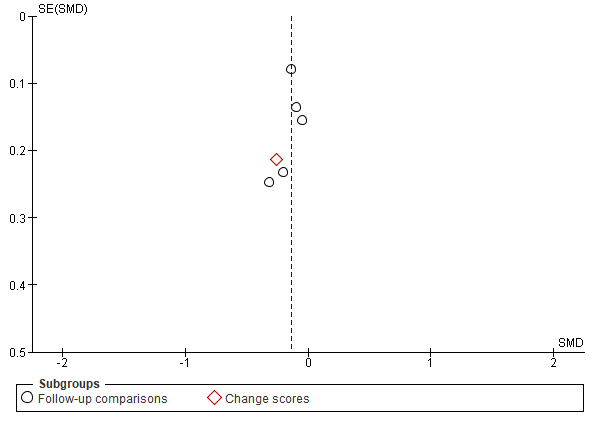


**Figure 6.** Funnel plot of comparison: 6 Self-efficacy, follow-up.


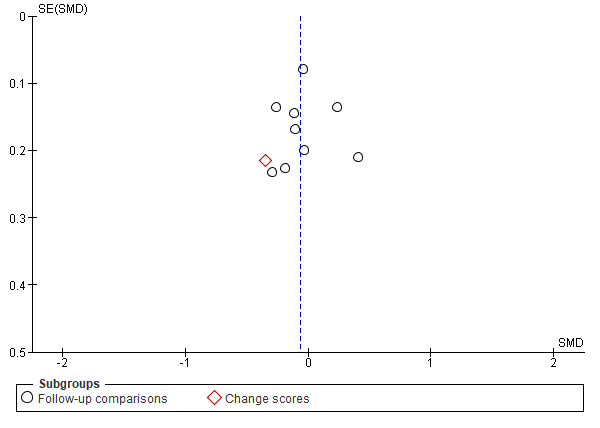


**Figure 7.** Funnel plot of comparison: 7 Pain intensity, follow-up.

**
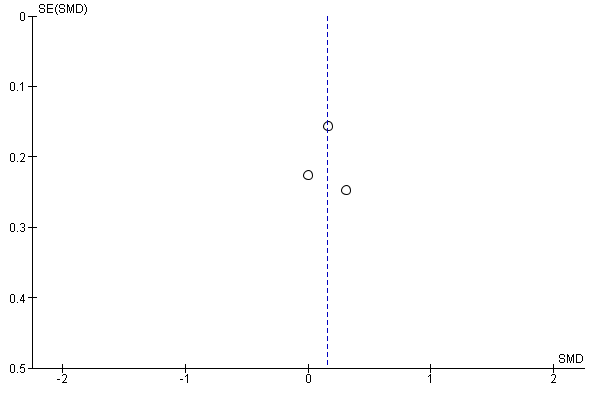
**

**Figure 8.** Funnel plot of comparison: 8 Physical activity, follow-up.
